# Vascularized liver-on-a-chip model to investigate nicotine-induced dysfunction

**DOI:** 10.1063/5.0172677

**Published:** 2023-12-27

**Authors:** Eric Wang, Melisa J. Andrade, Quinton Smith

**Affiliations:** 1 Department of Chemical and Biomolecular Engineering, University of California Irvine, Irvine, California 92697, USA; 2 Sue & Bill Gross Stem Cell Research Center, University of California Irvine, Irvine, California 92697, USA

## Abstract

The development of physiologically relevant *in vitro* systems for simulating disease onset and progression and predicting drug metabolism holds tremendous value in reducing drug discovery time and cost. However, many of these platforms lack accuracy in replicating the tissue architecture and multicellular interactions. By leveraging three-dimensional cell culture, biomimetic soft hydrogels, and engineered stimuli, *in vitro* models have continued to progress. Nonetheless, the incorporation of the microvasculature has been met with many challenges, specifically with the addition of parenchymal cell types. Here, a systematic approach to investigating the initial seeding density of endothelial cells and its effects on interconnected networks was taken and combined with hepatic spheroids to form a liver-on-a-chip model. Leveraging this system, nicotine's effects on microvasculature and hepatic function were investigated. The findings indicated that nicotine led to interrupted adherens junctions, decreased guanosine triphosphate cyclohydrolase 1 expression, impaired angiogenesis, and lowered barrier function, all key factors in endothelial dysfunction. With the combination of the optimized microvascular networks, a vascularized liver-on-a-chip was formed, providing functional xenobiotic metabolism and synthesis of both albumin and urea. This system provides insight into potential hepatotoxicity caused by various drugs and allows for assessing vascular dysfunction in a high throughput manner.

## INTRODUCTION

*In vitro* models have been leveraged for predicting drug toxicity, therapeutic responses, and disease modeling using stable cell lines and primary cell isolates.^1^ Despite major advances in biomedical sciences, these conventional models often pose a bottleneck, providing limited and physiologically inaccurate depictions of biological processes *in vivo*.^2^ Attempts to mimic *in vivo* processes *in vitro*, the combination of cells, embedded within biomimetic soft hydrogel matrices, have enabled the migration and remodeling of three-dimensional (3D) microenvironments, recapitulating aspects of physiologically relevant niches. While these models have been leveraged to phenocopy tissue microenvironments, triggering responses to stimuli similar to those found in human biology, many lack the biological and mechanical cues necessary to provide a true biomimetic response.^3,4^ Therefore, to further enhance biomimicry and physiological relevance, the incorporation of additional cues within the 3D *in vitro* models such as biochemical factors (growth factors, cytokines) and physical factors (fluid flow, mechanical strain) present in the native tissue allows for better replication of the tissue complexity, structural organization, and cellular crosstalk, which can lead to a more comprehensive understanding of disease progression and therapeutic response.^5,6^

Building upon the progress of these 3D *in vitro* models, the integration of microfluidics has emerged as a method to further enhance the biomimetic nature of these systems, with precise control over both biochemical and physical factors.^7^ In particular, leveraging microfluidics enables the development of a more accurate liver-on-a-chip, which is important for understanding drug metabolism and prevalent liver diseases such as Metabolic dysfunction-associated fatty liver disease (MAFLD). The modeling of the liver has been explored using different cell sources including primary cells,^8,9^ immortalized cell lines,^10,11^ and stem cell-derived hepatocytes.^12–14^ However, a common limitation of most of these models is the lack of a functional vasculature, which is crucial for cell–cell interactions and mimicking the liver’s physiological microenvironment. Models investigating vascularized liver-on-a-chip exist, primarily focus on the effects of heterotypic paracrine interactions in controlling hepatic stability, instead of leveraging these systems to investigate metabolic functions, including processing xenobiotics or drugs.^15,16^ Parallel efforts focused on developing microvasculature systems have led to the generation of perfusable capillary-like structures through cell–cell interactions,^17^ interstitial flow,^18,19^ and shear stresses.^20^ However, despite these advancements, the investigation of the combined effects of these vascularized systems on hepatic drug response and metabolism remains relatively understudied.^16^

These *in vitro* models are important in modeling various disease pathologies and their impact on homeostasis, particularly ones caused by drug addiction and abuse that could, otherwise, be avoided. Nicotine, widely known for its association with tobacco addiction, undergoes a significant portion of its metabolism in the liver, primarily by the enzyme cytochrome P450 (CYP) 2A6. Numerous studies have linked variable CYP2A6 activity, assessed through genotypic and phenotypic investigations, to altered risks associated with tobacco-related cancers and smoking behaviors. These behaviors include being a smoker, the number of cigarettes smoked, and the likelihood of smoking cessation. It is worth highlighting the inherent variability observed with *in vivo* studies examining nicotine metabolism and CYP2A6 activity, including large interethnic and interindividual differences. Similarly, studies involving human liver microsomes have demonstrated significant variations, exceeding 50-fold, in CYP2A6 mRNA, protein, and activity levels.^21^ To this end, understanding the complex metabolism of nicotine is of paramount importance, especially in the context of smoking and its effects on the vasculature. Consequently, there is a growing motivation to develop *in vitro* tools that can effectively mimic and study the impact of nicotine exposure.

In order to address this gap, a microfluidic model was developed, comprising of immortalized human hepatic spheroids (HepG2), human umbilical vein endothelial cells (HUVECs), and human dermal fibroblast (HDFs) to generate a vascularized liver-on-a-chip platform. Investigation into the formation of functional microvascular networks revealed that vasculogenic potential and extent of perfusability rely on the initial seeding concentrations of ECs and stromal cells. Utilizing the optimal conditions for microvascular network formation, hepatic spheroids were embedded within the vascularized microfluidic devices to produce a liver-on-a-chip capable of secreting albumin (ALB) and urea, indicative of hepatic functionality *in vitro*. Investigation into the ubiquitous drug nicotine on the vasculature was found to cause endothelial inflammation, as demonstrated through increased reactive oxygen species (ROS) and abrogated guanosine triphosphate (GTP) cyclohydrolase 1 expression. This inflamed state led to increased monocyte adhesion, decreased junctional cadherin expression, and impaired endothelial barrier function. Furthermore, it was found that when introduced to the vascularized liver-on-a-chip, nicotine caused a reduction in cytochrome P450 (CYP) 3A4 induction in response to acetaminophen (APAP), the active ingredient in many popular painkillers. Herein, the liver-on-a-chip model exhibited a possible antagonistic effect of the use of simultaneous drug use, with the potential to unravel the intricate mechanisms through which nicotine influences human vascular health, enabling a better understanding of the risks and potential interventions associated with smoking-related vascular diseases.

## RESULTS

### Control of heterotypic cell–cell interactions drives vascular assembly

**FIG. 1.**
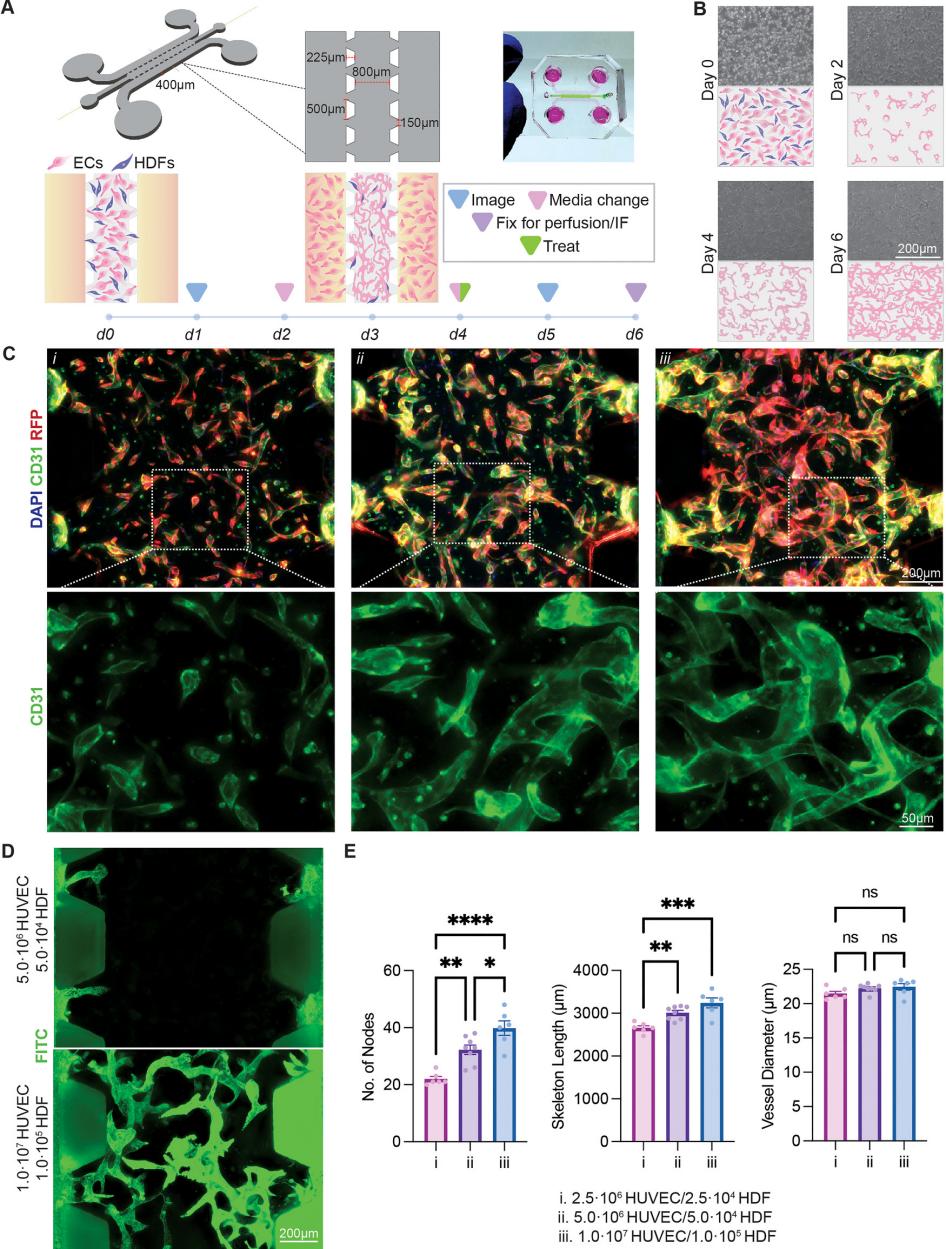
Formation of microvascular networks. (a) General schematic of microvascular networks and timeline. (b) Day-by-day microvascular network formation of 10 × 10^6^ HUVECs + 100 000 HDFs/mL. (c) Day 6 immunofluorescent max intensity images of (i) 2.5, (ii) 5, and (iii) 10 × 10^6^ HUVECs/mL with 100:1 ratio of HDFs. (d) Perfusion of 1 *μ*m FluoSpheres into the microvascular networks showing perfusability. (e) Quantification of key microvascular parameters. Each region of interest covers 0.11 mm^2^.

While previous studies using microvascular networks have extensively investigated the effect of the stromal to endothelial cell ratio in vessel formation, the effect of the cellular density itself on vasculogenesis has not been completely determined.^17^ To study this process, three parallel microfluidic channels, separated by angled geometric posts, which support extracellular matrix (ECM) patterning via surface tension, were fabricated using standard lithography techniques to support *de novo* vasculogenesis and angiogenic remodeling [Fig. 1(a)]. To drive vasculogenesis, human umbilical vein endothelial cells (HUVECs) and human dermal fibroblasts (HDFs) were seeded in the center microfluidic channel at varying seeding concentrations, maintaining a 100:1 ratio of ECs to fibroblasts, within an ECM containing 5 mg/mL bovine fibrinogen and 10% basement membrane extract (BME). The ratio of ECs to HDFs was carefully determined through a combination of literature reviews and iterative experiments. This ratio was optimized to strike a balance between beneficial heterotypic interactions while reducing excessive proliferation of HDFs, avoiding excessive fibrinolysis, and minimizing matrix remodeling by the fibroblasts. This approach was leveraged to ensure hydrogel stability throughout the duration of the experiment, as suggested by prior research.^23^ The device design permits the direct interaction of the ECM with media on both sides, reducing the diffusion length scale to ensure sufficient medium exchange. In addition, this allows for perfusability to be investigated by introducing beads or other small molecules to one medium channel and allowing them to flow through the vessels due to pressure and concentration gradients. On day 3 post-seeding, additional ECs were seeded within the two medium channels while tilting the devices to promote EC growth on the ECM, driving anastomosis with the preexisting vessels. With the highest initial seeding condition of 10 × 10^6^ HUVECs/mL and 100 000 HDF/mL, primitive vascular networks formed within 48 h of seeding, and interconnected networks were observed by day 4 [Fig. 1(b)]. Interestingly, at lower initial concentrations of 2.5 and 5× 10^6^HUVECs/mL with the same ratio of HDFs, the vascular networks followed similar trends but lacked the cellular density essential to form continuous lumen containing CD31 networks throughout the entire device by day 6 [Fig. 1(c)], thereby suggesting the importance of initial seeding density in the formation of microvascular networks.

Despite rudimentary network formation occurring under all the seeding conditions [Fig. 1(c)], only the highest seeding condition consisting of 10× 10^6^ HUVECs/mL was able to form microvascular networks capable of transporting fluorescent 1.0 *μ*m microspheres through the device, indicating the presence of continuous lumenal structures formed by the ECs [Fig. 1(d)]. Despite the CD31 expressing networks at a seeding concentration of 5× 10^6^ HUVECs/mL, the microspheres were unable to transport across the ECM containing channel. Instead, the beads could only be transported at most 100 *μ*m before clogging at a closed lumen. Similarly, the transport of beads was obstructed (data not shown) at 2.5× 10^6^ HUVECs/mL, further supporting the lack of a continuous network [Fig. 1(c)]. Utilizing AutoTube,^23^ a MATLAB-based program, the quantification of the microvascular networks revealed a direct correlation between the number of nodes and skeletal length with the initial cell seeding density [Fig. 1(e)]. Within the region of interest quantified, significantly fewer nodes were present in lower cell seeding densities, with 2.5, 5.0, and 10× 10^6^ HUVECs/mL having an average of 22.0 ± 0.89, 32.3 ± 1.68, and 39.8 ± 2.56 nodes, respectively. In addition, the observed skeletal length of 5× 10^6^ HUVECs/mL (3012.1 ± 54.8 *μ*m) and 10× 10^6^ HUVECs/mL (3242.2 ± 114.4 *μ*m) was significantly higher compared to 2.5× 10^6^ HUVECs/mL (2661.0 ± 48.7 *μ*m). Despite the changes in the skeletal structure, the mean diameter of the formed vessels (∼22 *μ*m) was independent of seeding conditions.

### Nicotine exposure results in adherens junctional irregularities

**FIG. 2.**
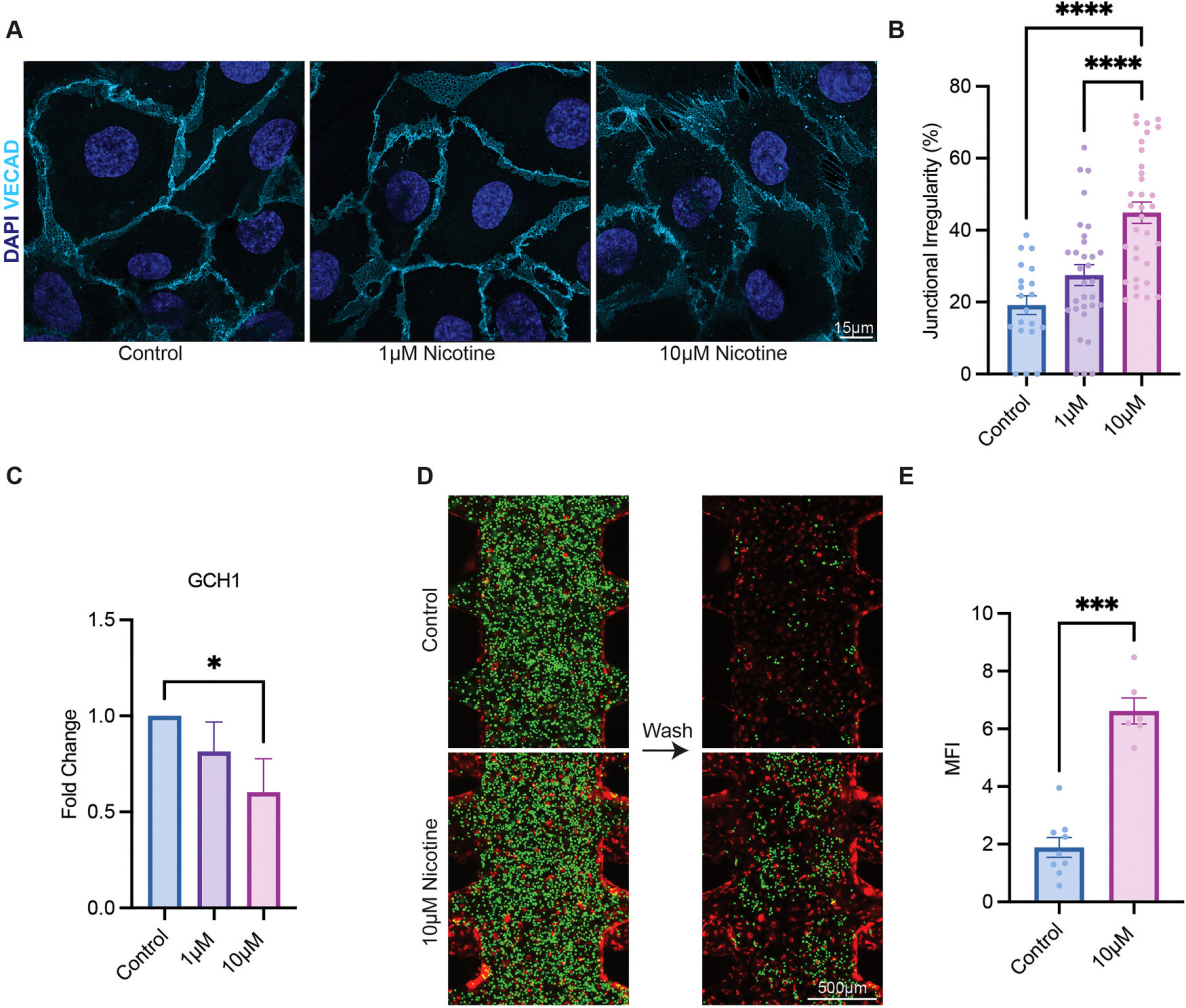
Endothelial monolayer response to nicotine. (a) Confocal images of HUVECs treated with 0, 1, and 10 *μ*M nicotine showing adherens junction irregularities. (b) Quantification of percent adherens junctions with irregularities including discontinuities and breaks. (c) Fold change in GCH1 of HUVECs treated with nicotine. (d) Monocyte adhesion assay utilizing calcein-AM labeled THP-1 monocytes (green), comparing control vs 10 *μ*M nicotine. (e) Quantification of MFI of THP-1 cells.

Investigation into irregularities in endothelial junctions can provide insights into potential barrier function integrity.^24^ To this end, junctional integrity was evaluated through immunofluorescence staining and quantifying vascular endothelial cadherin (VECAD) expression in a monolayer culture of HUVECs. VECAD expression and morphology were observed after a 48 h induction with exposure to 1 and 10 *μ*M nicotine for 48 h. Concentrations were determined through a combination of *in vitro*, *in vivo*, and clinical literature review, where plasma concentrations fall between 0.1 and 16 *μ*M.^25–29^ Observation of the cell–cell junctions revealed that nicotine exposure led to increased interrupted junctions and a decrease in reticular junctions, as seen by morphological changes in the cell–cell interfaces^30^ [Fig. 2(a)]. Previous studies found that decreased specialized reticular junctions led to a higher junctional permeability.^31^ A dose-dependent response to nicotine was observed by quantifying the entire cell–cell junction and determining the percentage of irregularities [Fig. 2(b)]. In the control, the percent of junctional irregularities remained relatively low (19.2 ± 2.6) vs the 1 *μ*M nicotine (27.5 ± 2.9). At the highest concentration of 10 *μ*M nicotine, disrupted junctions accounted for nearly the majority of cell–cell junctions, with 44.9 ± 3.0% interrupted. These findings indicate that nicotine exposure disturbs adherens junctions in endothelial cells.

### Nicotine downregulates GTP cyclohydrolase 1 in endothelial cells

GTP cyclohydrolase 1 (GCH1) is the rate limiting enzyme in the tetrahydrobiopterin (BH4) production.^32^ Decreased levels of BH_4_ lead to lowered endothelial nitric oxide synthase (eNOS), causing endothelial dysfunction. Previous studies have investigated the time dependency of GCH1 expression at a single dose of 1 *μ*M nicotine, finding decreasing levels over 48 h.^29^ To elucidate the dose-dependency of nicotine, 1 and 10 *μ*M nicotine were supplemented for 48 h to a monolayer of HUVECs. Compared to the control, 1 *μ*M nicotine decreased GCH1 expression to 0.81 ± 0.09, while 10 *μ*M nicotine led to a 0.60 ± 0.10 decrease, indicating a dose-dependency of nicotine treatment and GCH1 expression [Fig. 2(c)].

### Nicotine-induced endothelial inflammation enhances monocyte adhesion

Monocyte adhesion assays have been leveraged to determine the degree of endothelium inflammation in studies related to human immunodeficiency virus (HIV) infections and atherosclerosis.^36,37^ Through the expression of surface adhesion molecules, monocytes become tethered and are easily quantified. To determine the extent of endothelial inflammation caused by nicotine, a monolayer of HUVECs was cultured within the center microfluidic channel and treated for 48 h with 10 *μ*M nicotine. THP-1 monocytes, fluorescently labeled using calcein-AM, were introduced to the center channel and washed to remove after 5 min of incubation [Fig. 2(d)]. Compared to the control, the mean fluorescence intensity (MFI) of the monocytes adhered to nicotine-treated HUVECs increased more than threefold, from 1.9 ± 0.34 to 6.6 ± 0.45 [Fig. 2(e)], indicating that treating HUVECs with 10 *μ*M nicotine can induce an inflammatory response, rendering enhanced adhesion of monocytes to a monolayer of endothelial cells.

### Nicotine induces reactive oxygen species in microvascular networks

**FIG. 3.**
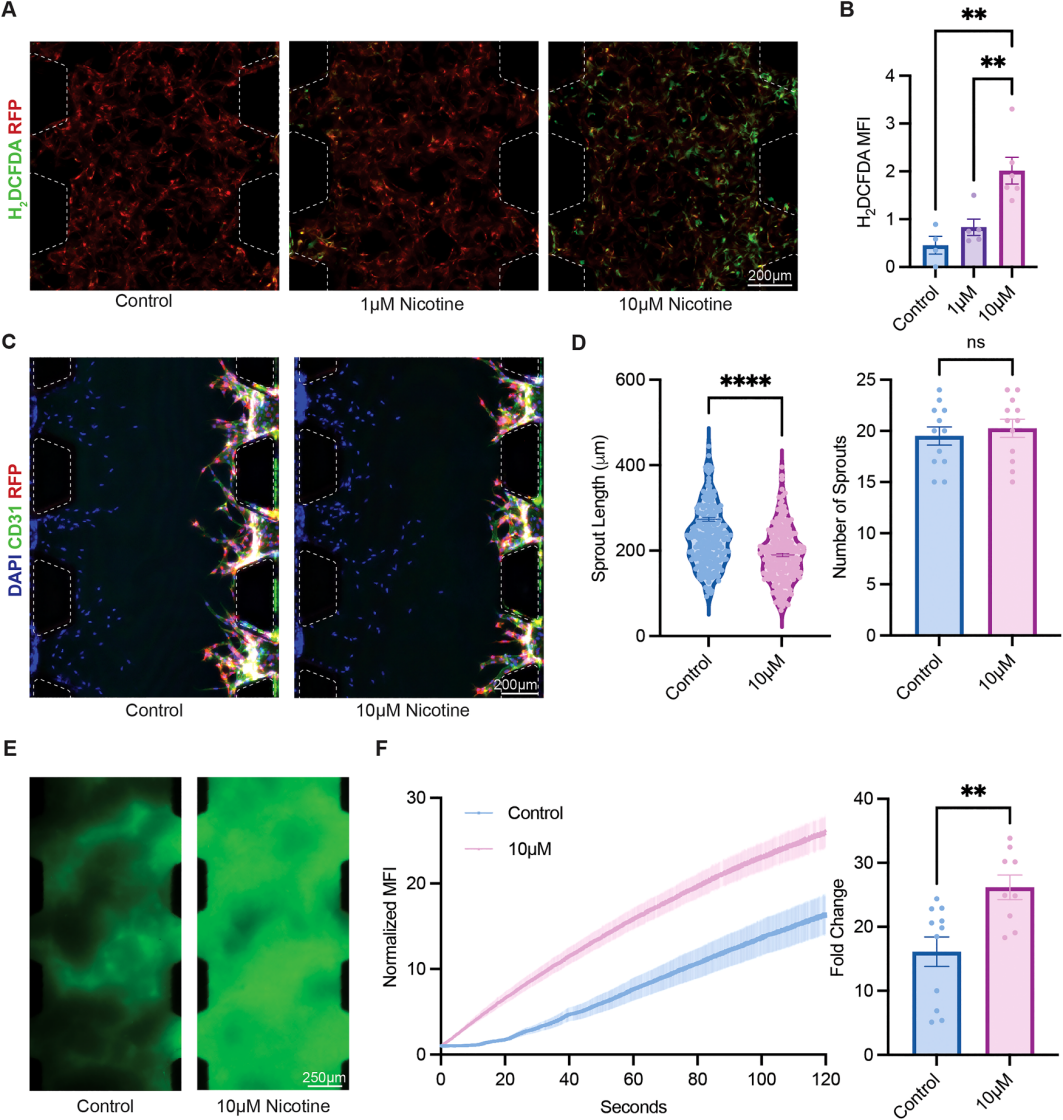
Microvascular network response to nicotine. (a) Reactive oxygen species (ROS) generation within microvascular networks detected by H_2_DCFDA after 48 h of nicotine exposure. (b) Quantification of ROS through H_2_DCFDA MFI. (c) Immunofluorescent images of angiogenesis assay on day 7. (d) Quantification of the sprout length and number of sprouts from angiogenesis assay. (e) Microvascular network perfusion of 70 kDa FITC-dextran after 120 s, showing leakiness. (f) Normalized mean fluorescence intensity (MFI) of extravasated FITC-dextran over 120 s and fold change after 120 s.

It is well-established that nicotine exposure results in endothelial inflammation, which, in turn, causes oxidative stress, leading to the increased production of reactive oxygen species (ROS). However, the extent of nicotine-induced ROS generation has not been investigated in biomimetic microvascular networks.^29,35^ Therefore, microvascular networks were formed within the microfluidic device to determine nicotine-induced ROS, utilizing the optimal initial seeding condition of 10 × 10^6^ HUVECs/mL with HDF at a 100:1 ratio for interconnected vascular networks. On day 4, when the microvascular networks were formed, the devices were treated with 1 and 10 *μ*M nicotine. After 48 h of treatment, H_2_DCFDA was utilized to detect the ROS species [Fig. 3(a)]. ROS production was strongly correlated with nicotine exposure [Fig. 3(b)]. Without nicotine exposure, the devices exhibited minimal ROS production (mean MFI of 0.45 ± 0.19) but significantly increased to a mean fluorescence intensity (MFI) of 0.83 ± 0.17 upon treatment with 1 *μ*M nicotine. Notably, with 10 *μ*M nicotine treatment, the intensity over control increased fourfold to an average MFI of 2.0 ± 0.28. These findings indicate a dose-dependent ROS production with increasing concentrations of nicotine in biomimetic microvascular networks.

### Nicotine alters angiogenic sprout length

Exogenous nicotine stimulates pathological angiogenesis through the stimulation of nicotinic acetylcholine receptors (nAChR).^36^ To study the effects of nicotine stimulation on endothelial angiogenesis *in vitro*, the existing platform was leveraged to create an angiogenic gradient by first introducing HUVECs to one side of acellular hydrogel loaded in the middle channel. Furthermore, the opposite medium channel was seeded with HDFs, supplemented with elevated serum levels and growth factors to generate an angiogenic promoting cytokine gradient, promoting angiogenic sprouting into the gel, thereby enabling sprout number and length measurement. 10 *μ*M nicotine was added to the medium channel containing the HUVECs to facilitate direct nicotine exposure. Both the control and the treated groups were able to form robust sprouts by day 7, as demonstrated by the immunofluorescence staining for CD31 [Fig. 3(c)]. While there was no significant difference in the number of sprouts in each region of interest (19.5 ± 0.88 for control, 20.3 ± 0.89 for treatment), the mean sprout length decreased in the treatment group, from 231.7 ± 4.49 to 189.4 ± 4.04 *μ*m, indicating impaired angiogenic remodeling [Fig. 3(d)].

### Nicotine exposure disrupts the barrier function of microvascular networks

Given the impaired angiogenic response, it was speculated that additional consequences of nicotine stimulation on vascular integrity could be interrogated. It was hypothesized that the observed impaired EC state would have an impact on barrier function. The barrier function of endothelial cells plays a crucial role in mediating what can permeate through blood vessels, regulating protein, ion, and water exchange. Abrogated barrier function can lead to infections or disrupt homeostasis.^37^ Previously, nicotine has been shown to reduce epithelial barrier function;^38^ however, this extent for endothelial cells has not been widely investigated. Microvascular networks were formed as previously described to investigate this, followed by 10 *μ*M nicotine treatment on day 4. A 70 kDa fluorescein isothiocyanate-dextran (FITC-dextran), representative of large biomolecules such as albumin, which is the most abundant protein found in the blood, was introduced to the medium channels, driving a differential for fluid to flow through the assembled vascular networks and imaged using a timelapse microscope.^39–43^ Compared to untreated controls, an increase in the FITC-dextran concentration in the hydrogel of nicotine-containing devices was observed, indicating that the microvascular networks were unable to retain the FITC-dextran intravascularly due to leakier junctions, hence demonstrating a reduced barrier function phenotype [Fig. 3(e)]. Following the trend found in the qualitative results, the normalized MFI of FITC-dextran in regions of interest (ROIs), selected as extravascular regions containing only the hydrogel, increased over the observation period under both conditions; however, at 120 s, 10 *μ*M nicotine-treated samples led to over a 60% higher fold change compared to untreated samples (26.2 ± 1.92 vs 16.1 ± 2.30) [Fig. 3(f)]. These results further quantify the extent that nicotine can reduce the barrier function of microvascular networks.

### Embedding of hepatic spheroids within microvascular networks

**FIG. 4.**
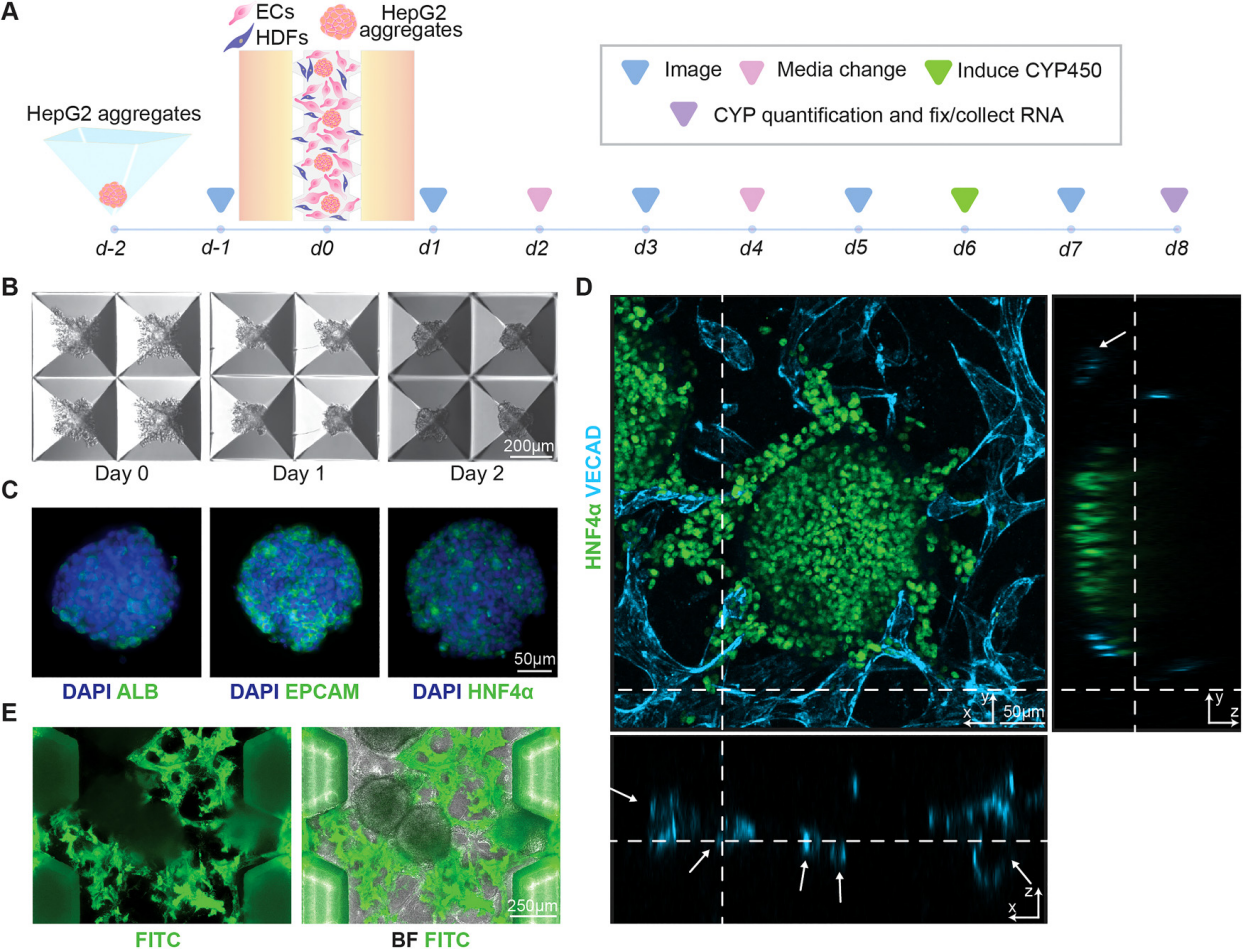
Formation of the vascularized liver-on-a-chip model. (a) General timeline of the formation of the liver-on-a-chip. (b) Day-by-day brightfield image of aggregation of HepG2 cells using Aggrewells. (c) Immunofluorescent images of the hepatic spheroid stained with various hepatic markers. (d) Orthogonal confocal image of vascularized hepatic spheroids with open lumen indicated with arrows. (e) Perfusion of 1 *μ*m FluoSpheres into vascularized liver-on-a-chip showing interconnected perfusable networks.

The microfluidic system was extended to include hepatocytes, generating a liver-on-a-chip to study the effects of nicotine in a biomimetic vascularized system [Fig. 4(a)]. Immortalized HepG2 hepatocyte cells were aggregated using Aggrewells, a commercially available pyramidal-shaped microwell, clustering after 24 h and forming tight spheroids after 48 h of culture [Fig. 4(b)]. Despite previous studies utilizing HepG2 spheroids, limited data demonstrate retained hepatic gene expression.^43,44^ Here, the expression of hepatic markers albumin (ALB), hepatocyte nuclear factor 4 alpha (HNF4α), epithelial cell adhesion molecule (EPCAM), and alpha-1 antitrypsin (A1AT) remained, evidenced through immunofluorescence staining. Additionally, BODIPY staining indicated that HepG2 cells retained lipid-storage ability, confirming that the aggregated HepG2 cells retain their hepatic-like status [Fig. 4(c) and Fig. S3 in the supplementary material^87^].

To develop a vascularized liver-on-a-chip model, aggregated HepG2 cells were seeded concurrently under optimized HUVEC and HDF seeding conditions, resulting in interconnected, perfusable networks. Upon confocal analysis, open luminal structures were found in VECAD expressing cell networks, denoting functional microvasculature surrounded by HNF4α-expressing spheroids [Fig. 4(d) and Fig. S4 in the supplementary material^87^]. To further support that an open and interconnected lumen was present, fluorescent microspheres were introduced to the medium channels, which could be transported fully through the device, even reaching the spheroids [Fig. 4(e)]. Collectively, the described liver-on-a-chip platform supports fluid transport and cellular arrangement, mediating heterotypic signaling between vascular and hepatic cells, and promoting a more accurate and physiological phenotype.

### Vascularized liver-on-a-chip enhances functional hepatic outputs

**FIG. 5.**
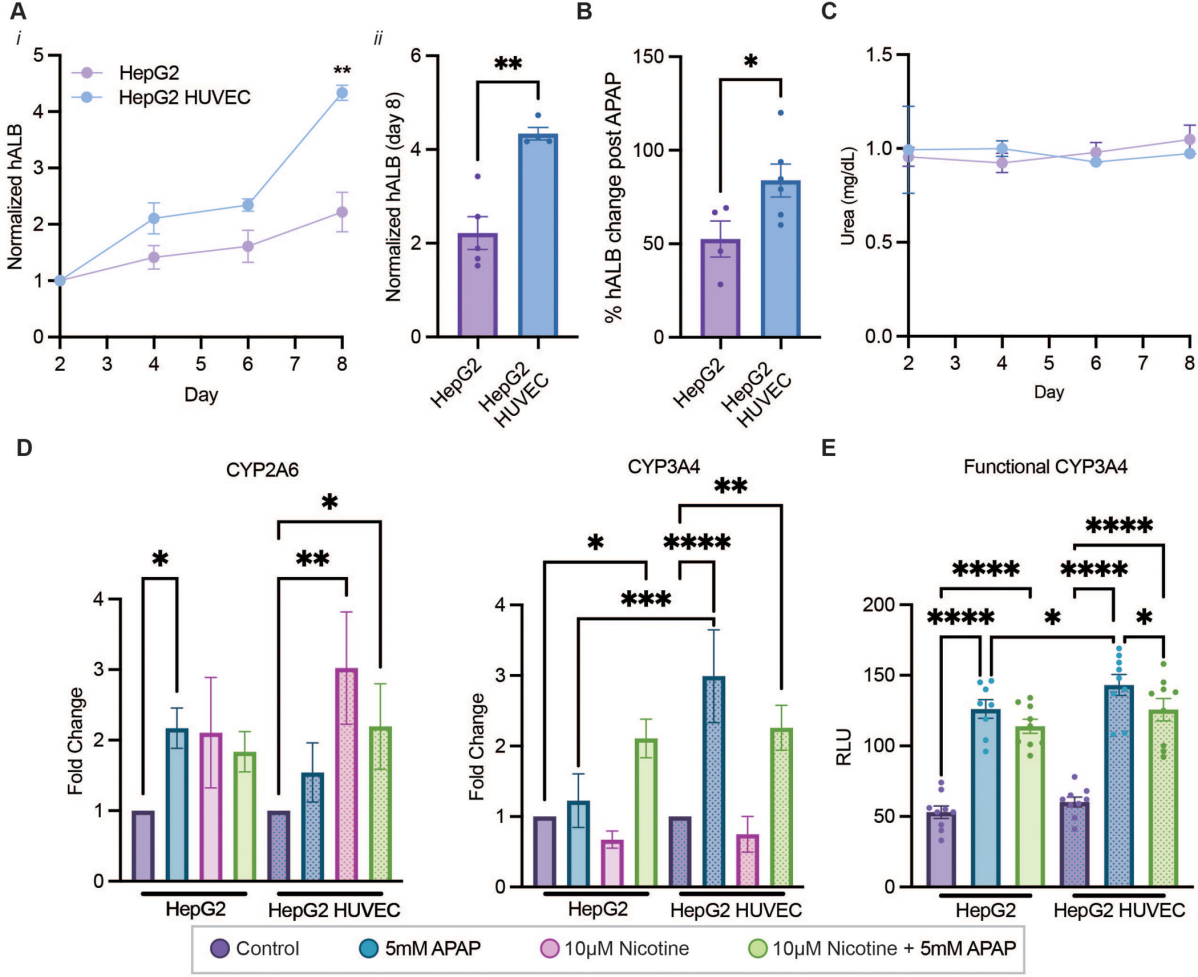
Vascularized liver-on-a-chip model provides functional outputs. (a) Secreted albumin from microvascular networks, non-vascularized, and vascularized liver-on-a-chip. (i) The normalized (to day 2) time course values are shown along with (ii) day 8 values. (b) Change in albumin values after 48 h exposure to 5 mM APAP when compared to the corresponding control. (c) Secreted urea from non-vascularized and vascularized liver-on-a-chip. (d) Fold change in transcriptional CYP2A6 and CYP3A4 of the liver-on-a-chip. (e) Functional CYP3A4 metabolism with non-vascularized and vascularized liver-on-a-chip when treated with 5 mM APAP and/or 10 *μ*M nicotine.

One critical function of hepatocytes is serum protein biosynthesis. Albumin secretion by the liver is not only clinically utilized as an indicator of hepatic health but is responsible for the transport of hydrophobic molecules and maintains oncotic body pressure. For example, in diseased livers with cirrhosis, albumin levels are drastically reduced.^45^ To this end, the basal functionality of the liver-on-a-chip system was evaluated by measuring albumin secretion. In the vascularized liver-on-a-chip model, 3967.5 ± 463.6 ng/mL of albumin was secreted at the first medium change on day 2, with increasing albumin output over the 8-day time course ending with secretion of 14 105.6 ± 463.6 ng/mL produced from day 6 to day 8 (Fig. S5 in the supplementary material^87^). Compared to the vascularized model, HepG2-only devices produced more albumin, with a day 2 reading at 8096.0 ± 2115.0 ng/mL and day 8 17 070.5 ± 718.9 ng/mL. Upon normalizing to day 2 readings, it was found that adding vasculature leads to a quicker increase in albumin secretion, consistent with previous reports,^46^ suggesting that heterotypic interactions promote hepatic functionality compared to monoculture conditions as previously described [Fig. 5(a)i].^47,48^ This increase in normalized albumin can be seen clearly on day 8, with an almost twofold increase over the HepG2-only model [4.34 ± 0.13 vs 2.22 ± 0.35, Fig. 5(a)ii]. Interestingly, when 5 mM acetaminophen (APAP) was administered to these devices for 48 h, it was seen that albumin secretion was less affected in the vascularized system when compared to its control (83.9 ± 8.8% of control), while a more significant decrease was found in the HepG2-only system (52.6 ± 9.6% of control) [Fig. 5(b)]. This is thought to be due to the recapitulation of the paracrine interactions between ECs and hepatocytes, which assists in liver regeneration and supports liver function.^16,49^ Nitrogen handling was also evaluated in the liver-on-a-chip platform by measuring urea, a hepatocyte metabolite of ammonia filtered and excreted by the kidneys.^50^ This by-product of catabolism is detrimental in ureotelic animals such as mammals as it can accumulate without a method to excrete.^34^ Approximately 1 mg/dL of urea was present in the spent media for both the treatment and control devices, indicating that the vasculature within the devices does not significantly impact urea synthesis [Fig. 5(c)].

### Expression of transcriptional levels of CYP3A4 and CYP2A6 on liver-on-a-chip

The hepatotoxicity of acetaminophen (APAP), a commonly used analgesic and antipyretic drug, is primarily metabolized by CYP3A4 and depends on the balance between the formation of its hepatotoxic metabolite and the glutathione synthesis rate in the liver. Factors such as ethanol pretreatment in animal studies and chronic alcoholism in patients have been shown to increase the activity of cytochrome P450 enzymes,^51^ a key mechanism in which the liver metabolizes xenobiotics, driving acute acetaminophen toxicity.^52^ Moreover, animal experiments have demonstrated compelling evidence linking nicotine to hepatic steatosis and MAFLD, manifesting as heightened oxidative stress, elevated levels of hepatic triglycerides, an increased incidence of hepatocellular apoptosis, inactivation of AMPK, and activation of its downstream target, acetyl-coenzyme A-carboxylase (ACC).^57^ Overall, studying drug–drug interactions in the context of nicotine can provide a deeper understanding of how smoking impacts liver metabolism. To this end, the biomimetic vascularized liver-on-a-chip system was utilized to demonstrate the effects of nicotine stimulation on the response of hepatocytes to APAP.

Vascularized and non-vascularized devices were treated with 10 *μ*M nicotine, 5 mM APAP, and a combination of the two for 48 h. With only 10 *μ*M nicotine treatment, both vascularized (0.75 ± 0.25) and non-vascularized (0.67 ± 0.12) CYP3A4 levels fell; however, CYP2A6 levels increased (3.02 ± 0.80 for vascularized, 2.11 ± 0.78 for non-vascularized). The vascularized model provided a much more pronounced response compared to without. With the introduction of 5 mM APAP, higher CYP3A4 levels were found compared to their respective controls, with a significant change found when comparing the vascularized to the non-vascularized system (2.99 ± 0.65 for vascularized, 1.25 ± 0.38 for non-vascularized) [Fig. 5(d)]. CYP2A6 levels also increased with this treatment (1.54 ± 0.42 for vascularized, 2.17 ± 0.28 for non-vascularized). The combined treatment returned similar transcriptional levels of CYP3A4 compared to the 5 mM APAP treatment (2.26 ± 0.32 for vascularized, 2.11 ± 0.27 for non-vascularized). CYP2A6 levels also closely followed the trend found with the combination treatment (2.20 ± 0.61 for vascularized, 1.84 ± 0.29 for non-vascularized). These results indicate the transcriptional response of CYP3A4 and CYP2A6 to common xenobiotic treatment. Despite only being transcriptional, the presence of both basal and inducible CYP450s in the liver-on-a-chip system demonstrates the benefit of aggregation and embedding of HepG2 cells within a vascularized ECM, as previous works have found low to no CYP450 expression in these cells.^54^

### Induction of functional cytochrome P450 3A4 in liver-on-a-chip

CYP3A4 activity for the control vascularized device returned a mean relative luciferase intensity (RLU) of 60.2 ± 3.55 [Fig. 5(e)]. When induced with 5 mM of APAP for 48 h, CYP3A4 activity increased twofold to a RLU of 143.1 ± 7.40. Compared to the non-vascularized model, the control had a CYP3A4 activity RLU of 53.0 ± 4.35, and the induced returned an RLU of 126.1 ± 6.58. These indicate that the liver-on-a-chip can metabolize drugs, with vasculature significantly increasing CYP3A4 activity over the non-vascularized counterpart. When 10 *μ*M nicotine was co-administered with 5 mM APAP for 48 h, the CYP3A4 activity of the vascularized device fell to 125.7 ± 7.97 from 143.1 ± 7.40 RLU and the non-vascularized model fell to 113.9 ± 4.98 from 126.1 ± 6.58 RLU when compared to the solo administration of 5 mM APAP. This indicates that, despite CYP3A4 not being the enzyme responsible for nicotine metabolism, reduction in the CYP3A4 activity may occur. In addition, these results corroborate the transcriptional findings, supporting that the proposed liver-on-a-chip is capable of metabolizing xenobiotics.

## DISCUSSION

The formation of microvascular networks is a crucial step toward forming more physiologically relevant lab-on-a-chip systems for drug development and disease modeling. This work assessed the initial seeding concentration of endothelial and supporting stromal cells, determining differences in the microvascular skeletal morphology and perfusability. High cellular densities were found to drive vasculogenesis, allowing interconnected networks to form. Thereafter, the effects of nicotine were investigated, combining findings from 2D monolayer studies and 3D biomimetic microvascular networks. Upon exposure to nicotine, irregularities in junctions, increased monocyte adhesion, higher ROS production, inhibited angiogenesis, and increased permeability were observed. Incorporating hepatic spheroids in microvascular networks produced a vascularized liver-on-a-chip, possessing key morphological characteristics and functionality, including albumin production, nitrogen fixation, and cytochrome P450 activity. Toward the end goal of producing systems for *in vitro* research, this work presents a more biomimetic lab-on-a-chip, where the combination of vasculature and hepatic spheroids allows cellular crosstalk through direct interactions and diffusible factors as found in the human body.

Extensive research on the formation of microvascular networks has been published, investigating the effects of flow-induced stress, cytokine gradients, and cellular compositions.^5,18,19,22^ However, most of these studies lack a systematic investigation into simple methods to change microvascular geometries, relying on more complex and less accessible methods. By increasing initial cellular concentration, it was possible to promote continuous lumen containing vascular networks throughout the device while maintaining the supporting cell ratio. Importantly, by retaining a low ratio of HDFs, the secreted vascular endothelial growth factor (VEGF) and other cytokines from the fibroblasts can promote and stabilize the vascular assembly without the concern of extensive proliferation and ECM degradation.^55,56^ This provides for a stable system that enables investigation into various angiogenic pathologies induced by drug use or environmental factors. Importantly, it must be noted that while the initial seeding density directly influences vessel assembly within a specified range as investigated; over a certain range, it may attain a steady state at which there may be minimal or no changes to the vascular network morphology owing to critical homo- and heterotypic interactions.^57,58^

To study the impact of nicotine on endothelial cells, a combined approach involving monolayer HUVEC culture and the microfluidic vascular system was employed. This strategy aimed to enhance the fidelity of the described *in vitro* model for representing relevant biological processes. Observations revealed that the exposure of HUVEC monolayers to nicotine over a 48 h period increased junctional irregularities, particularly evident in VECAD expression. These findings suggest a potential mechanistic link between nicotine exposure, VECAD irregularities, and subsequent effects on tight junctions. This insight contributes to our understanding of the cellular impacts of nicotine on endothelial function within the described microvascular platform. With the depletion of VECAD, despite other junctional proteins being expressed, cellular permeability was increased,^59^ suggesting that the irregularities and decrease in reticular junctions caused by nicotine exposure decrease barrier function in capillaries. This was validated through the investigation of microvascular networks. When pre-existing vessels were treated with nicotine, there was a significant increase in the permeability of 70 kDa FITC-dextran. This relationship between nicotine, increased permeability, and endothelial dysfunction has been observed previously in clinical settings; however, a lack of representative *in vitro* models prevented this phenomenon from being demonstrated *in vitro*.^60,61^ Further investigation into permeability of wider ranges of molecules with differing hydrophilicity, length, and charge to simulate currently utilized drugs will allow a better understanding of the mechanism behind the increased permeability.^39–43^

In addition to permeability, nicotine increased monocyte adhesion in monolayers of HUVECs. This correlated with previous work utilizing serum derived from smokers.^62^ The increased adhesion onto the endothelial cells may be caused by inflammatory cytokines mediating upregulation β_1_ and β_2_ integrin interactions with the monocytes.^63^ Despite the lack of significant changes in the gene expression of inflammatory markers such as ICAM1 and VCAM1 present at 48 h, mirroring past research,^64,65^ it has been found that other possible inflammatory endothelial ligands may be responsible for the observed increased monocyte adhesion.

Elucidating the gene expression of GCH1 after 48 h exposure to nicotine in the setting of the monolayer showed significant downregulation for the highest concentration of 10 *μ*M. These findings are in line with previous *in vitro* studies due to nicotine interfering and suppressing human antigen R (HuR) cytoplasm migration, leading to impeded BH_4_ synthesis.^29^ While not investigated in this study, hypoxic conditions (5% O_2_) have been shown to further downregulate GCH1. To determine the downstream effects in a biomimetic setting, nicotine exposure to pre-existing microvascular networks increased ROS at 48 h of exposure. While these findings agree with the literature, they have not previously been shown in biomimetic microvascular networks. The decreased BH_4_ synthesis, alongside the increase in superoxide anions, causing BH_4_ to oxidize to an incompetent BH_2_ form, has been found to decouple endothelial nitric oxide synthase (eNOS), leading to endothelial dysfunction.^66^ Nitric oxide (NO) reportedly reduced leukocyte aggregation and adherence,^67^ complimenting previous results, wherein nicotine enhances monocyte adhesion through decreased GCH1 expression. Furthermore, the resulting angiogenic sprout length decrease, when cultured in the medium containing 10 *μ*M nicotine, was possibly caused by the reduction in NO, leading to lower endothelial proliferation and matrix remodeling.^67–70^

To extend the scope of the microvascular system, the combination of the microvascular networks and HepG2 spheroids enabled the ability to form a liver-on-a-chip. In correlation with previous research,^43,44^ upon aggregation, the HepG2 spheroids expressed the characteristic hepatic and epithelial markers. Important for drug metabolism studies, the aggregation of HepG2 cells has been found to upregulate CYP450-mediated xenobiotic metabolism, and albumin secretion.^71^ Thereafter, these spheroids were embedded within the microfluidic devices, with or without the perfusable microvascular networks, shown by open luminal structures in confocal imagining alongside the ability to transport 1 *μ*m microspheres. When co-cultured with HUVECs, C3As (a derivative of HepG2) secreted more albumin than in monoculture, indicative of higher hepatic synthesis function due to heterotypic interactions.^72^ Despite the lower absolute albumin over the hepatic-only models, the vascularized models showed increased normalized albumin when compared to the initial reading on day 2, suggesting that endothelial interactions assist HepG2s in recovery from initial seeding. Urea was present in both the vascularized and non-vascularized, indicating functional ammonia conversion, further supporting hepatic synthesis functionality.^46^ This model is not without limitation as the vascularized system lacked the native architecture found in physiology. Instead, this system aimed to recapitulate heterotypic paracrine interactions between the ECs and hepatic spheroids, found to play crucial roles in liver regeneration in physiological settings.^16,49^

To utilize and assess the functionality of this liver-on-a-chip, APAP, a commonly used pain and fever reducer, was administered on day 6 after established microvascular networks were formed. It was found that CYP3A4, the main enzyme responsible for the metabolism of APAP, could be induced.^54^ With the combined dosage of APAP and nicotine, the CYP3A4 activity was present, however, for both the vascularized and non-vascularized, which showed trends of decreased activity. This possible antagonistic effect of simultaneous drug use has not been shown either *in vivo* or *in vitro*; however, it proves to be a point of further interest, as nicotine is mainly a substrate of CYP2A6, not CYP3A4.^73^ Previous *in vivo* studies have found that long term smoking in rats caused inhibited CYP3A1 activity, the rat analog to human CYP3A4;^74,75^ however, this effect has not been found *in vitro* prior to this work, nor has it been shown for short term effects.

The CYP450 gene family expression from HepG2 cells has been reported to be minimally expressed, with previous works engineering the cell line to express various CYP450.^54,76^ As mentioned, the aggregation of HepG2 cells has shown to upregulate the CYP450 family;^71^ however, the impact of co-culture alongside ECs has not been thoroughly investigated. When looking at CYP3A4, a higher fold mRNA change was shown in response to the 5 mM treatment of APAP with the addition of HUVECs. A similar trend was shown with the mRNA expression of CYP2A6, with a higher fold change when compared to the absence of HUVECs. These results are indicative that, in addition to the aggregation of HepG2, the presence of endothelial cells also upregulate various CYP450s, including CYP3A4 and CYP2A6 at a transcriptional level, allowing for a more accurate model on drug metabolism. Of importance, when looking at transcriptional changes in the cocultured system, RNA was isolated from the system as a whole, including the ECs and HDFs, and then normalized to glyceraldehyde 3-phosphate dehydrogenase (GAPDH) and TATA-box binding protein (TBP). Due to this, the fold changes seen in CYP2A6 and CYP3A4 may be artificially lowered in the vascularized system due to ECs and HDFs having negligible inducible transcriptional CYP450 and a larger normalization coefficient (expression of systemic GAPDH and TBP).^77^ It must be noted that it has been found that mRNA changes have not always led to functional CYP450 changes; however, this work showed similar trends in the functional CYP3A4 quantification.^78^ In combination with the increased normalized rate of albumin production as a proxy for hepatic synthesis function, these results provide potential evidence of merit in the vascularized liver-on-a-chip.

The use of primary and immortalized cell lines here allowed for the fabrication of a high throughput vascularized liver-on-a-chip, giving insight into drug-induced microvascular dysfunction and xenobiotic metabolism. However, even with the aggregation and co-culture, the ability to fully mimic vasculature and hepatocytes is limited with these cultured cells. These effects were seen in this model, as a higher dose of APAP was needed to induce a functional CYP3A4 response. In order to combat this and give rise to more physiologically accurate systems, stem cell-derived endothelium^79–81^ and hepatocytes are of great interest. Stem cell-derived endothelial cells enable the generation of tissue-specific vasculature, furthering the ability to mimic specific niches,^82^ allowing differentiation toward liver sinusoidal endothelial cells (LSECs) possessing a fenestrated phenotype, which is often lost when culturing primary LSECs *in vitro*.^83–85^ Furthermore, stem cell-derived hepatocytes have shown to have a degree of CYP450 functionality, with the ability to be derived from patient samples posing as a more tractable cell source than HepG2 cells.^86^

## MATERIALS AND METHODS

### Microfluidic chip fabrication

The microfluidic master was fabricated through standard soft lithography processes, utilizing SU-8 2100 (Kayaku) to form a 400 *μ*m high layer on a silicon wafer. The wafer was then exposed to UV light through a photomask and developed, giving rise to the master mold. Polydimethylsiloxane (Sylgard 184, Dow) was cast on top of the master and bonded irreversibility to a glass bottom plate using oxygen-plasma treatment for 60 s (Harrick). The channels of the devices were then coated with poly-L-lysine (0.01% w/v, 84 kDa, Millipore) and glutaraldehyde (1.0%, Sigma) to promote gel adhesion.

### Cell culture

Human umbilical vein endothelial cells (HUVECs) expressing RFP (Angio-Proteomie) were cultured in endothelial growth medium 2 (EGM2, PromoCell) supplemented with 10% fetal bovine serum (FBS) (Gibco). These cells were passaged or harvested for use at 70%–80% confluence until passage 10. Human dermal fibroblasts (HDFs) (PromoCell) were cultured in fibroblast growth medium 2 (FGM2, PromoCell) until confluent and then passaged or utilized until passage 15. THP-1 cells (ATCC) were grown in RPMI-1640 (Gibco) supplemented with 10% FBS in suspension cultures, maintaining below 3.0 × 10^6^ cells/mL by diluting with fresh media. The cells were passaged at confluence (1.0 × 10^8^ cells in the flask). HepG2 cells (ATCC) were cultured on a collagen type-1 coated flask (Corning) in Dulbecco’s modified Eagle’s medium (DMEM)/F12 (Gibco) supplemented with 10% FBS, 1× non-essential amino acids (Gibco), and 1× L-glutamine (Gibco). These cells were passaged at confluence. All cells were grown at 37 °C and 5% CO_2_.

### Formation of microvascular networks in microfluidic chip

HUVECs and human dermal fibroblasts (HDFs) (PromoCell) were harvested and seeded at various concentrations in the middle channel of the microfluidic device within a hydrogel containing 5 mg/mL bovine fibrinogen (Thermo), 10% basement membrane extract (Bio-Techne), and polymerized with bovine thrombin (Thermo). After three days, additional HUVECs were seeded on the side channels.

### Angiogenesis assay

Using the same microfluidic chip, an acellular gel was seeded in the center channel at the same composition. HUVECs were seeded in the top medium channel at a density of 5 × 10^6^ HUVECs/mL, and then, the device was tilted for 10 min to allow cells to attach to the gel. After 24 h, HDFs were seeded at 200 000 cells/mL in the lower medium channel, along with supplemental 10% FBS (Gibco) and 50 ng/mL of VEGF (PeproTech) to promote angiogenesis. Nicotine was added to the top medium channel with the HUVECs. On day 7, these devices were fixed and stained, and then, ImageJ was used for quantification.

### Immunofluorescence staining

Samples were fixed overnight in 4% paraformaldehyde (Santa Cruz) on an orbital shaker and then washed with phosphate-buffered saline (PBS). Then, samples were blocked and permeabilized for an hour using a PBS buffer containing 3% bovine serum albumin (Sigma) and 0.5% Triton X-100 (Sigma). Primary antibodies (Thermo) were diluted 1:500 with blocking buffer and incubated overnight at 4 °C on an orbital shaker. The primary antibodies were then removed, and the samples were washed overnight in PBS. Corresponding secondary antibodies (Thermo) were diluted 1:500 with blocking buffer, along with phalloidin (1:200, Thermo) and 4',6-diamidino-2-phenylindole (DAPI) (1:1000, Thermo), and the sample was returned to the shaker overnight.

### Microvascular quantification

Samples were imaged using Keyence BZ-x800, using a z-stack to capture the entire height of the sample. Max intensity projection of regions of interest was utilized for quantification. Using AutoTube (ETH Zürich), the key parameters of the vessels were quantified. Perfusability and permeability were investigated by introducing 1 *μ*m FluoSpheres (Thermo) or 70 kDa FITC-dextran (Santa Cruz) to the medium channels while taking a timelapse.

### Vessel functionality assays

To assess the functionality of the microvascular network, nicotine, a known agonist of nicotinic acetylcholine receptors, was introduced. H_2_DCFDA (Invitrogen) was utilized to detect reactive oxygen species. The mean fluorescence intensity for regions of interest was quantified using ImageJ. Quantification of key vascular parameters was performed utilizing AutoTube under different treatment conditions. A MATLAB script was written to quantify the mean fluorescent intensity outside of the vessels over a time course, as an analog to the concentration of FITC-dextran permeating through the vessels.

### Monocyte adhesion assay

To determine the extent of endothelial inflammation caused by nicotine, HUVECs were cultured in a monolayer within the center channel of the microfluidic channels and then treated with nicotine for 48 h. From here, THP-1 monocytes cultured in RPMI 1640 (Gibco) supplemented with 10% FBS (Gibco) were labeled with 10 *μ*M calcein-AM (Invitrogen) to fluorescently label live cells. After a 5 min incubation, the THP-1 cell suspension was removed and washed with PBS. Comparing the MFI of images taken before and after allowed to see the adhesion of these monocytes.

### Formation of hepatic spheroids

Aggrewell 400 (STEMCELL Technologies) was used to aggregate HepG2 cells, following the manufacturer protocols, at a density of 500 cells per microwell. On day 2, the spheroids were harvested from the wells with gentle pipetting, ensuring not to dissociate the spheroids.

### Liver-on-a-chip functionality assays

To form the liver-on-a-chip model, the harvested spheroids were mixed with the EC gel precursor and then injected into the microfluidic chip. The medium was collected every other day for the quantification of secreted albumin (Bethyl) and nitrogen fixation (Invitrogen). To induce cytochrome P450, acetaminophen (Sigma) and nicotine (Sigma) were mixed directly into the culture medium on day 6. After a 48 h induction, CYP3A4 (Promega) was quantified following the manufacturer instructions.

### RNA isolation and RT-PCR

To isolate the RNA, the middle channel of the microfluidic device was cut out using a blade and then scraped into TRIzol (Thermo). Total RNA was isolated using a PureLink^TM^ RNA mini kit (Thermo) following the manufacturer instructions. The RNA quality and concentration were assessed using Nanodrop. cDNA synthesis was performed on the total RNA using the High-Capacity cDNA Reverse Transcription Kit (Thermo). Quantitative real-time polymerase chain reaction (RT-PCR) was performed using Taqman probes on a QuantStudio 3 Real-Time PCR System (Thermo). The gene expression was normalized to housekeeping genes, GAPDH and TBP, and the respective fold changes were determined.

### Statistical analysis

Data were analyzed in GraphPad Prism 9, plotting the mean and standard error of the mean (SEM). For comparisons of two groups, Mann–Whitney t-tests were utilized to determine significance. For larger datasets, one-way analysis of variance (ANOVA) was utilized, making multiple comparisons between groups.

## CONCLUSION

In this study, a systematic approach was taken to fabricate perfusable microvascular networks, which in conjunction with hepatic spheroids formed liver-on-a-chip, providing critical insights into physiological responses to commonly used xenobiotics. It was found that nicotine caused impaired barrier function, junctional irregularities, and interfered with the eNOS signaling pathway. The functional ability to synthesize albumin and urea was assessed in the vascularized and non-vascularized liver-on-a-chip. Furthermore, these systems demonstrated both transcriptional and functional abilities to induce CYP450 responses to drugs, thereby providing better physiological approximations with *in vitro* systems, allowing for higher throughput and spatiotemporal resolution compared to *in vivo* systems.

## SUPPLEMENTARY MATERIAL

See the supplementary material^87^ for additional quantification and images.

## Data Availability

The data that support the findings of this study are available from the corresponding author upon reasonable request.
